# Pharmacist prescribing in hospital inpatient settings: what works, for whom, why and in what circumstances—a realist review protocol

**DOI:** 10.1136/bmjopen-2026-117974

**Published:** 2026-05-11

**Authors:** Dayana El Nsouli, Stephen Timmons, Claire Anderson, Adam Gordon, Naoko Arakawa

**Affiliations:** 1 Pharmacy Department, Royal Derby Hospital, Derby, England, UK; 2 Division of Pharmacy Practice and Policy, University of Nottingham School of Pharmacy, Nottingham, England, UK; 3 University of Nottingham Business School, Nottingham, England, UK; 4 Wolfson Institute of Population Health, Queen Mary University of London, London, UK; 5 Academic Centre for Healthy Ageing, Barts Health NHS Trust, London, UK

**Keywords:** Pharmacists, Prescriptions, Inpatients

## Abstract

**Abstract:**

**Introduction:**

Pharmacist prescribing has been introduced to alleviate pressures on hospital services and improve timely access to treatment. However, implementation in inpatient settings remains highly variable, with pockets of excellent practice alongside areas where prescribing roles are limited or absent. Traditional effectiveness reviews have demonstrated positive impacts of pharmacist prescribing on clinical and service outcomes yet offer limited insight into how contextual conditions and underlying mechanisms interact to produce results in complex hospital inpatient environments. This realist review aims to develop and refine programme theory (PT) explaining how pharmacist prescribing in hospital inpatient settings works, for whom, why and in what circumstances, with particular attention to the factors that support or constrain successful implementation.

**Methods and analysis:**

The review will follow Pawson’s six-stage realist synthesis process andRealist And Meta-narrative Evidence Syntheses: Evolving Standards guidance, moving iteratively between theory development, searching, selection, data extraction and synthesis. A multidisciplinary stakeholder advisory group and patient and public involvement group will work alongside the review team to feedback on the scope, refinement of context-mechanism-outcome configurations (CMOCs) and implementation recommendations. Initial programme theories will be developed and then refined using evidence from formal searches of MEDLINE, Embase, CINAHL and Scopus alongside grey literature. Data will be extracted into a descriptive spreadsheet and coded in NVivo using deductive, inductive and retroductive approaches to identify, test and refine CMOCs. The final output will be a refined PT and practical recommendations to inform design, implementation and scaling of pharmacist prescribing roles in hospital inpatient care with attention to equity and acceptability for patients and multidisciplinary teams.

**Ethics and dissemination:**

Ethics approval is not required for this realist review as it involves secondary analysis of published articles and grey literature. Dissemination will include peer-reviewed publications, presentations to pharmacy departments and professional bodies, as well as co-produced accessible materials with patient and public groups to support knowledge mobilisation. The review protocol has been registered on PROSPERO.

**PROSPERO registration number:**

CRD420261283633.

**STRENGTHS AND LIMITATIONS OF THE STUDY**

- This study uses realist methods to explain how, why, for whom and in what contexts pharmacist prescribing works in hospital inpatient settings.
- Includes a variety of evidence sources, combining peer-reviewed studies and grey literature, to inform the development and refinement of programme theory made up of context-mechanism-outcome configurations.
- Incorporates ongoing input from expert multidisciplinary stakeholder groups and involves patients and the public throughout.
- Restricts evidence to UK, English-language documents from 2006 onwards which may limit transferability to other health systems.
- Relies on interpretive judgement in configuring mechanisms and contexts, which may introduce subjectivity.

## Introduction

 Pharmacist prescribing has been strategically used to address the ever-increasing pressures on the National Health Service (NHS), improve medicines optimisation and enhance patient access to timely treatment.^1^ These pressures are global as health systems face challenges in costs, efficiency, access and effectiveness.^2^ Evidence from a range of settings suggests that pharmacist prescribing can improve disease control, reduce hospital utilisation and enhance the safety and appropriateness of medicines use, although the magnitude and consistency of these benefits vary by context.^3^,^6^

Within the UK, the pharmacy profession has progressively expanded its scope to include independent prescribing to all newly registered pharmacists to support workforce transformation.^7^ All pharmacists who graduated from GPhC accredited pharmacy programmes and get into the UK register from 2026 would be independent prescribers. This workforce transformation is paralleled with professional standards for hospital pharmacy explicitly encouraging the integration of pharmacist prescribers into relevant care pathways.^8^ Despite these developments, implementation in hospital inpatient settings remains uneven, with local variation in role design, governance, digital infrastructure, with pockets of excellent practice.^9^ While we know through traditional effectiveness studies that pharmacist prescribing has had a positive impact on the health service, we do not know what explains this variation. In a complex intervention like this, understanding what works, for whom, how and in what circumstances necessitates a realist approach that considers complex organisational environments.^10 11^

A realist review is a suitable methodology and approach to evidence synthesis as it seeks to unpack how outcomes are generated through context-mechanism-outcome configurations (CMOCs).^12^ Using the Realist And Meta-narrative Evidence Syntheses: Evolving Standards (RAMESES) reporting standards to ensure rigorous and transparent application of this methodology, a realist review of hospital pharmacist prescribing can generate explanatory programme theory (PT) that is directly useful for policymakers, educators and service leaders looking to design and implement these roles.^13^ The review therefore seeks to answer: How does pharmacist prescribing in hospital inpatient settings work, for whom, why and in what circumstances? Additional sub-questions include:

- What mechanisms lead to the success of pharmacist prescribing in inpatient settings?
- What contextual factors shape the mechanisms and corresponding outcomes?

## Methods

This review protocol has been registered in PROSPERO (CRD420261283633). The review commenced in April 2026, with database searches and screening planned to be completed by November 2026, and synthesis and write up by April 2027.

### Design and approach

A realist review will be conducted to develop and refine PT explaining how, why, for whom and in what circumstances pharmacist prescribing in hospital inpatient settings produces specific outcomes. The review will follow the six-stage process described by Pawson *et al*.^14^ This approach uses theory-driven evidence synthesis to identify the CMOCs that explain how complex interventions work such as pharmacist prescribing in inpatient hospital settings. It will follow an iterative, theory-driven logic of enquiry to continuously refine a developing PT.

### Overview

**Figure 1:**
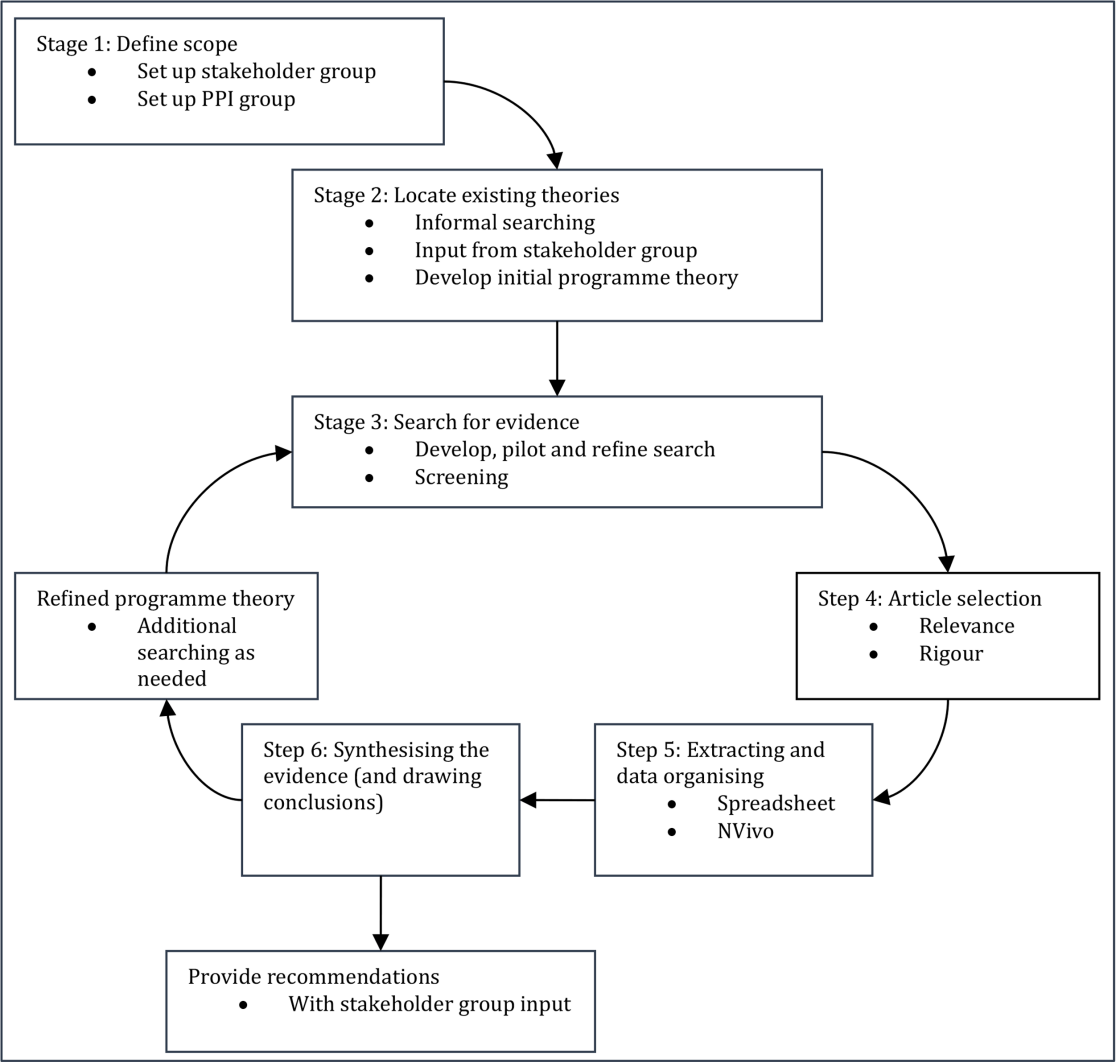
Six-stage flowchart of realist review process. PPI, patient and public involvement.

The review will progress through a series of interconnected stages that move iteratively between theory development, evidence searching, data extraction and synthesis. These stages are illustrated in figure 1, which summarises the planned process from initial scoping to a refined PT. This fluidity in the approach reflects realist principles which allow movement between stages as new findings may require prompt refinement of the review focus, additional searches or re-examination of already included data.^13^

### Stage 1: define scope and advisory groups

Realist review guidance emphasises the value of a stakeholder group to help shape the scope, focus and provide feedback on the iterative PT versions.^13^ Their involvement also strengthens the relevance and interpretive validity of the CMOCs as empirical data underlying the PT. Therefore, a multidisciplinary stakeholder advisory group will be established at the outset. It will comprise hospital pharmacist independent prescribers, members of the wider multidisciplinary team such as physicians, nurses and allied health professionals, service managers, education leads, and representatives from professional and regulatory bodies involved in prescribing policy and guidance. The group will meet at key points in the review to include refining and prioritising review questions and drafting initial PT; providing experiential and contextual insight in interpreting CMOCs; sense-checking proposed mechanisms against everyday practices and organisational realities; and co-producing recommendations that are feasible and acceptable within hospital settings.

In addition, a patient and public involvement (PPI) group will provide the patient voice throughout the whole review. The group will include patients who have had experience of being in the care of pharmacist prescribers as well as carers. These members will contribute to clarifying which outcomes matter most to patients, refining and commenting on the iterations and PT drafts, as well as sense-checking whether proposed mechanisms and contexts resonate with their experiences. PPI members will also be invited to collaborate on co-production of recommendations, as well as any patient-facing outputs and dissemination plans. In line with the National Institute of Health and Care Research’s (NIHR) strategy on equality, diversity and inclusion, both groups will purposively reflect variation in gender, age, ethnicity and background to include voices from underserved communities.^15^

### Stage 2: locate and develop initial theories

This next stage will focus on an early ‘theory-gleaning’ phase which draws on diverse theoretical, empirical and policy sources to generate candidate explanations in a CMOC format.^16^ It includes locating and articulating different theories about how pharmacist prescribing is expected to work or fail in hospital inpatient settings. These theories will then act as a scaffold for subsequent searching and analyses in the next phases as well as a working hypothesis that is tested and refined as the review progresses.

A preliminary, informal search will be undertaken to identify relevant theories and concepts of pharmacist prescribing in hospital and possibly to include closely related roles such as (other) non-medical prescribers. The types of sources expected to be included are empirical studies, service evaluations, policy, professional guidance, opinion papers and grey literature such as reports and frameworks. Attention will be paid to both explicit theoretical propositions, such as interprofessional working and role identity, as well as more implicit assumptions embedded in descriptions of service models and implementation efforts.^10^

Within this stage, the initial PT that results from the searches will be structured using a CMOC heuristic. The context will focus on features of the hospital and specific wards such as service type, staffing models, digital infrastructure, culture and multidisciplinary working. The intervention will be defined as pharmacist prescribing models, care pathways, scope of practice and clinical responsibilities. Candidate mechanisms are expected to include trust, rapport, role legitimacy, self-efficacy and perceived competency which may be triggered differently across different contexts. Finally, anticipated outcomes could include prescribing volume, readiness, patterns, timeliness and acceptability by wider teams and patients. The draft PT will then be presented to the advisory groups for discussion, critique and refinements. Their feedback will be used to expand, clarify or re-prioritise theories and identify additional issues that warrant exploration in subsequent review stages.

### Stage 3: iterative searching

During this stage, a formal search strategy will be developed to identify empirical and theoretical evidence relevant to the initial PT. Realist principles will be adhered to by being inclusive of sources that can inform or challenge emerging CMOCs.^12^ Searches will initially focus on pharmacist prescribing in hospital and may expand to analogous evidence from non-medical prescribers where underlying mechanisms may be transferrable. Electronic databases where searches will be conducted include MEDLINE, Embase, CINAHL and Scopus using a combination of database-specific headings and keywords. An example MEDLINE search strategy is in online supplemental appendix 1, and sample keyword searches in the format of Population, Context is in table 1.

**Table 1:** Sample keyword search strategy

Population	Pharmacist prescribing, independent prescribing pharmacist
Context	Hospital, inpatient, ward-based

The search will be inclusive of all study designs such as qualitative, quantitative, service evaluations, audits and implementation studies. Grey literature will also be searched for relevant data such as NHS reports, hospital policies and professional or regulatory guidance. Searches will be limited to English language only, and UK documents published from May 2006 onwards reflecting the period when independent prescribing authority was granted to pharmacists in the UK.^17^ Forward and backward citation tracking of papers, paralleled with searching reference lists, will be used to identify additional sources that can contribute to theory development.

### Stage 4: select and appraise evidence

After all records have been imported into Covidence reference software, they will be screened by title/abstract and then full text against a predefined eligibility criteria listed in table 2. This criterion has been developed to identify documents that can meaningfully inform theory building. Where eligibility is uncertain, documents will be retained and their relevance judged at full text with notes to their potential contribution in specific CMOCs. Two reviewers will independently screen titles, abstracts and full texts independently, and a third reviewer will check a 10% sample at each stage to identify and resolve any discrepancies. Reasons for exclusion at full text will be recorded and presented in a Preferred Reporting Items for Systematic reviews and Meta-Analyses (PRISMA) diagram.

**Table 2:** Screening eligibility criteria

Inclusion	Exclusion
Describe, evaluate or theorise pharmacist prescribing	Pharmacist roles that do not involve independent prescribing
Hospital inpatient setting	Outpatient, community or primary care setting
Provide data that can contribute to understanding contexts, mechanisms and/or outcomes of pharmacist prescribing	
UK based, English language, published May 2006 onwards	

Guided by the principles of realist methodology, inclusion and exclusion decisions will be based on relevance and rigour in relation to PT development and testing. Relevance refers to whether data can inform, refine or challenge CMOCs.^18^ Rigour concerns the plausibility and trustworthiness of the data in how they were generated and reported.^19^ Plausible data will be those that are sufficiently believable to justify their influence on the PT.^20^ Trustworthy data is judged as dependable and reliable through the appropriateness and transparency of data collection and analysis methods.^20^ Any documents that contain highly relevant but not as rigorous data may still be included if they offer valuable insights though their contributions will be weighted cautiously during the analysis.

### Stage 5: extract, code and configure CMOCs

For each included document, data extraction will be in two separate categories: descriptive and analytical. Descriptive data will be collected on an Excel spreadsheet which will record study characteristics such as citation details, hospital and ward type, study aim and design, participants, description of pharmacist role and prescribing model and results. The full text will also be imported into NVivo to allow for analytical coding of relevant excerpts that contribute to CMOCs.

Analytical coding will combine deductive, inductive and retroductive approaches in line with realist methodological guidance.^21^ Deductive codes will be derived from the initial PT developed in stage 1. Inductive codes are from the included documents which will allow new concepts and unanticipated CMOs to emerge from the data. Retroductive coding will be used to infer underlying generative mechanisms that may not be explicit in the included sources but can plausibly explain how or why observed outcomes took place.^22^ To enhance consistency and transparency, a 10% sample of extracted and coded data will be independently checked by a second reviewer with discrepancies discussed and coding framework refined as needed with the wider team.

### Stage 6: refine PT and implementation recommendations

In this final stage, CMOCs that have been formed from across published studies will be compared, contrasted and synthesised to identify patterns in how contexts trigger mechanisms to generate certain outcomes. Analytical techniques such as juxtaposition, reconciliation, adjudication and consolidation will be used to refine and where necessary reconfigure PTs.^21^

They will be brought together to refine overarching PTs to explain pharmacist prescribing in inpatient hospital settings. These refined versions will be discussed with the advisory groups to test their face validity, practical credibility and usefulness. Stakeholders will be invited to sense-check proposed mechanisms and contextual explanations, identify gaps or misinterpretations, and consider implications for workforce, education and service design. Through these discussions, the advisory groups with the review team will co-produce a set of implementation recommendations that specify how pharmacist prescribing roles might be best introduced, supported and sustained under different contextual conditions. Attention will be paid to equity, patient safety, cultural competence and acceptability by the wider multidisciplinary team and patients.

## Equality, diversity and inclusion

In addition to EDI (equality, diversity and inclusion) principles being adhered to during the advisory group recruitment process, the NIHR guidance on inclusive research will also be reflected in the data extraction and synthesis phase.^15^ The team will actively consider how contexts, mechanisms and outcomes may differ across hospital types (eg, teaching tertiary centres vs district general hospitals), ward specialities and inpatient populations (eg, older people, people with multimorbidity and those with limited health literacy). Explicit reporting on evidence gaps related to equity will be made.

## Ethics and dissemination

Ethics approval is not required as this is a protocol for a realist review which involves secondary analysis of existing published and grey literature. Stakeholder and PPI groups will follow institutional good-practice guidance on informed participation and confidentiality.^23^ Dissemination strategy will follow an integrated approach that aligns with NIHR’s guidance on dissemination and knowledge mobilisation.^24^ The aim will be not only to share findings but to support active use. Knowledge mobilisation principles will inform early and ongoing engagement with local stakeholder groups so that PTs and recommendations can be shaped, tested and adapted in real-world settings. In addition, planned outputs include peer-reviewed publications of the realist review, presentations to pharmacy departments at regional sites, as well as briefings to professional and regulatory bodies. Co-produced and accessible infographics will also be developed with PPI group for patients and public.

## Patient and public involvement

A PPI group was first engaged during the funding application stage for this NIHR Doctoral Clinical Academic Fellowship. Members helped identify and prioritise questions about pharmacist provision of patient care. Their lived experience informed the refinement of the overall research question in this realist review and then the subsequent realist evaluation. The PPI group will continue to be involved throughout the review in refining iterations of the PT.

## Supplementary material

10.1136/bmjopen-2026-117974 online supplemental file 1
