# Supplementary material for: Pharmacist prescribing in hospital inpatient settings: what works, for whom, why and in what circumstances—a realist review protocol
Source: BMJ Open. 2026 May 11;16(5):e117974. doi: 10.1136/bmjopen-2026-117974 (PMC13182349; doi:10.1136/bmjopen-2026-117974)
Supplement: online supplemental file 1 [file bmjopen-16-5-s001.docx]

**Appendix 1: Example MEDLINE Search Strategy**

| 1 | exp Pharmacists/ |
| --- | --- |
| 2 | exp Pharmacy Service, Hospital/ |
| 3 | exp Drug Prescriptions/ |
| 4 | 1 or 2 |
| 5 | 3 and 4 |
| 6 | (pharmacist* adj4 prescrib*).ti,ab,kf. |
| 7 | "independent prescrib*".ti,ab,kf. |
| 8 | "non-medical prescrib*".ti,ab,kf. |
| 9 | 5 or 6 or 7 or 8 |
| 10 | exp Hospitals/ |
| 11 | exp Inpatients/ |
| 12 | (inpatient* or in-patient*).ti,ab,kf. |
| 13 | ((hospital* or ward* or "critically ill" or "critical care" or "intensive care" or "high dependency" or CCU or ICU or HDU) adj4 patient*).ti,ab,kf. |
| 14 | ((acute or non-acute or "critical care" or "intensive care" or "high dependency" or "secondary care" or "tertiary care" or CCU or ICU or HDU) adj4 (setting* or ward* or unit* or hospital* or service*)).ti,ab,kf. |
| 15 | 10 or 11 or 12 or 13 or 14 |
| 16 | 9 and 15 |
| 17 | limit 16 to English language |
